# Assessment of changes in genetic transcriptome in nasal epithelial cells exposed to ozone-aged black carbon and pollen allergen by high-throughput transcriptomics

**DOI:** 10.1186/s13223-021-00553-2

**Published:** 2021-05-22

**Authors:** Yuhui Ouyang, Ying Li, Zhaojun Xu, Yusan An, Luo Zhang

**Affiliations:** 1 Department of Allergy, Beijing Tongren Hospital, Capital University of Medical Science, Beijing, 100730 China; 2 Department of Otolaryngology Head and Neck, Beijing Tongren Hospital, Capital University of Medical Science, Beijing, 100730 China; 3 Beijing Key Laboratory of Nasal Diseases, Beijing Institute of Otolaryngology, No. 17, HouGouHuTong, Dongcheng District, Beijing, 100005 China; 4 Department of Environmental Medicine, Quanzhou Medical College, Quanzhou, 362011 Fujian China; 5 Department of Biochemistry, Interdisciplinary Graduate School of Medicine and Engineering, University of Yamanashi, Yamanashi, 409-3898 Japan

**Keywords:** Ozone-aged black carbon (O_3_BC), Pollen allergen, Differential gene expression, Gene Ontology, High-throughput RNA sequencing, Human nasal epithelial cell (hNECs), Inflammation, KEGG pathway analysis, Transcriptome

## Abstract

**Background:**

Air pollution may be associated with increased airway responsiveness to allergens in allergic rhinitis (AR). Ozone-aged environmental black carbon (O_3_BC) is an important constituent of atmospheric particulate matter (PM), for which the mechanisms underlying its effects have not been fully elucidated in AR. The objective of the present study was to determine the O_3_BC and pollen-induced alterations in the transcriptome in human nasal epithelial cells (hNECs) in vitro.

**Methods:**

hNECs from nasal epithelial mucosal samples of healthy individuals undergoing nasal surgery (turbinoplasty or septoplasty) were established as air–liquid interface (ALI) cultures and exposed to O_3_BC, pollen, or a combination of O_3_BC+ pollen. Changes in cell viability were analyzed by fluorescence and changes in the transcriptome by high-throughput RNA sequencing (RNA-seq). Several differentially expressed genes were verified by reverse transcription-quantitative polymerase chain reaction (RT-qPCR). Enrichment analysis, based on Gene Ontology (GO) and Kyoto Encyclopedia of Genes and Genomes (KEGG) database, was performed to determine major biological functions and pathways involved.

**Results:**

Exposure to ≥ 50 μg/ml O_3_BC or 25 μg/ml O_3_BC+ 200 μg /ml pollen significantly decreased cell viability of the hNECs compared to control (p < 0.05) or 25 μg/ml O_3_BC alone (p < 0.05); whereas exposure to pollen alone did not alter cell viability at any concentration investigated. High-throughput RNA sequencing analysis indicated that there was significant difference in gene expression between pollen or O_3_BC alone and O_3_BC+ pollen exposed cells. Exposure to 200 μg/ml O_3_BC was associated with hypoxia stress response GO terms, whereas exposure to 25 μg/ml O_3_BC+ 200 μg/ml pollen was associated with inflammatory response GO terms; including regulation of neutrophil migration and chemotaxis, macrophage differentiation and chemotaxis, mast cell activation, and phagocytosis. KEGG pathway analysis indicated the top 10 upstream regulators to be IL1B, CSF1, CCL2, TLR2, LPL, IGF8, SPP1, CXCL8, FCER1G and IL1RN; of which expressions of inflammation-related genes IL1B, CSF1 and FCER1G were significantly increased.

**Conclusion:**

O_3_BC and pollen allergen combined exposure may induce innate immune and allergic inflammation in hNECs, and therefore potentially exacerbate the symptoms of AR in affected individuals.

## Introduction

Allergic rhinitis (AR) is a common inflammatory disease of the nasal mucosa, the prevalence of which has markedly increased over the past three decades and currently affects 10% to 40% of the population worldwide [1]. Epidemiologic studies have correlated the increase in AR in China with an increase in industrialization and air pollution over the last two decades [2]. Some studies have suggested that air pollution might induce an increase in airway responsiveness to allergens and increase bioavailability of airborne allergens [3]. In this context, as a typical outdoor aeroallergen, pollen affects about 30–58% of AR patients [4]. Studies investigating the effect of nitrogen dioxide (NO_2_) and sulphur dioxide (SO_2_), two major fossil fuel-derived air pollutants, have demonstrated that these increase the allergenicity of pollen [5], thus possibly aggravating or inducing AR in susceptible individuals.

Black carbon (BC) produced by the incomplete combustion of fuel is an important constituent of atmospheric particulate matter (PM) and is the second-largest contributor to global warming after carbon dioxide (CO2) in terms of direct forcing [6]. When emitted into the atmosphere, BC undergoes an aging process during which its particle morphology, chemical features, and redox activity may change, resulting in a component known as ozone-aged BC (O_3_BC) [7]. In the present study we have investigated the alterations in the transcriptome in human nasal epithelial cell (hNECs) exposed to O_3_BC and pollen allergen in vitro.

## Materials and methods

### Isolation and cultivation of human nasal epithelial cells (hNECs)

Nasal epithelial tissues were obtained from seven patients undergoing nasal surgery (turbinoplasty or septoplasty). Nasal polyps and the nasal tissues from patients with allergies, indicated by a positive serum allergen-specific IgE test to a variety of allergens (including *Humulus*, mold, *blattella*, dog dander, cat dander, house dusts, dust mites, *Artemisia annua*, common ragweed, and trees), or other chronic epithelial diseases were excluded. None of the patients had other systemic diseases or had received glucocorticoids or antibiotics within 3 months before the study. The study protocol was approved by the Ethics Committee of Beijing Tongren Hospital, and all patients provided written informed consent prior to any samples being taken for investigation.

Freshly obtained nasal mucosal samples were washed in phosphate buffer saline (PBS) with 200 U/mL penicillin and 50 mg/mL streptomycin, and then incubated overnight at 4 °C in 0.1% pronase (Protease XIV; Sigma-Aldrich, St. Louis, MO, USA) in Dulbecco's modified eagle media (DMEM) culture medium to enzymatically digest the tissue. Following incubation, the separated epithelial cells were collected and washed by centrifugation at 100*g* for 5 min and re-suspension in fresh DMEM. The washed cells were seeded at a concentration of 1 × 10^6^ cells on porous membrane inserts (Corning® Transwell polycarbonate membrane inserts, 0.4 μm; 6.5 mm diameter; Corning Inc., N.Y., USA) coated with 150 μl collagen I (66 ng/ml; Sigma-Aldrich, St. Louis, Mo., USA), and cultured at 37 °C in 5% CO2 in air atmosphere. Once the cultures had reached 70–80% confluence by day 4, the culture medium was removed from the inserts, and bronchial epithelial growth medium (BEGM): DMEM (1:1) medium was added to the basolateral side (insert wells) to differentiate cells. The cell cultures were assessed for transepithelial resistance (TER) using Millicell-ERS Volt-Ohm Meter (Millipore, Temecula, CA, USA). When the TER of individual cultures exceeded 2000Ω × cm^2^, the cultures were established as air–liquid interface (ALI) cultures, and subsequently used to assess the effects of exposure to O_3_BC and pollen.

### ***O***_***3***_*** BC and pollen preparation and exposure***

Ozone-aged black carbon (O_3_BC), which is consistent with black carbon in the real environment [8], was obtained from State Key Joint Laboratory of Environmental Simulation and Pollution Control, College of Environmental Sciences and Engineering, Peking University. The size of particles was approximately 30 nm. Prior to use, the O_3_BC was suspended in deionized water at a concentration of 2 mg/ml, and the suspension was mixed using a vortex mixer. The suspension was then sonicated for 10 min in a bath sonicator (TA4905, Tamagawa Seiki, Nagano, Japan) to achieve uniformity.

*Artemisia annua* (also known as annual mugwort or annual wormwood) pollen were collected from Beijing and aqueous protein extracts of the pollen were prepared by resuspending 2 g of pollen grains in 35 mL PBS buffer (0.14 M NaCl, 2.7 mM KCl, 7.8 mM Na2HPO4, 1.5 mM KH2PO4) and shaking for 12 h at 4 ℃. The supernatants were collected for estimation of the protein concentration, using the bicinchoninic acid (BCA) method (Thermo Fisher Scientific, Carlsbad, CA, USA). The pollen protein was then dissolved in deionized water at a concentration of 10 mg/ml.

The cells on Transwell membranes of ALI cultures were washed twice with sterile PBS, and the supernatant was removed. The membranes were exposed to O_3_BC at concentrations of 12.5, 25, 50, 100, 200 and 300 μg/ml O_3_BC; to pollen at concentrations of 50, 100, 200 and 300 μg/ml pollen; or a combination of 12.5, 25, 50, 100, 200, or 300 μg/ml O_3_BC and 200 μg/ml pollen for 24 h. Following exposure, the medium from the basolateral compartment of the Transwell was transferred to a 1.5 ml vial and stored at –20 °C until further analysis. The cells were trypsinized off from the membrane, lysed in RLT buffer (RNeasy mini kit, Qiagen, Ven- lo, The Netherlands), and stored at − 80 °C until further analysis.

### Cell viability

Cytotoxicity effects of BC and pollen in hNECs were determined using the Cell Counting Kit-8 (CCK8 CK04-500T Dojindo, Japan), according to manufacturer’s protocol. Cells (5 × 10^4^/well in 96-well plates) were exposed to either O_3_BC at concentrations ranging from 12.5 to 300 μg/ml, pollen at concentrations ranging from 50 to 300 μg/ml, or a combination of both O_3_BC and pollen for 24 h at 37 °C. 10 μL CCK8 solution was added to each well and the cells were incubated for a further 2 h at 37 °C. At the end of this incubation, the fluorescence of individual wells was determined at 450 nm using fluorescence microplate reader (Hitachi, Ltd., Tokyo, Japan). For each experiment, cultured cells from three different batches were assessed in triplicate (n = 9 cultures/exposure).

### High-throughput RNA sequencing (RNA-Seq)

For the transcriptomic studies, ALI-hNECs were treated with 25 μg/ml O_3_BC ± 200 μg/ml pollen for 24 h at 37 °C in CO_2_ incubator. At the end of incubation the hNECs were harvested and total RNA was extracted from the cells using RNeasy mini kit 147 ((Qiagen, Germany)). All RNA samples were stored at − 80 °C until use. For each experiment, three different batches of cultured cells were used and assays were performed in triplicate (n = 9 assays/exposure).

Paired-end libraries were synthesized by using the TruSeqTM RNA Sample Preparation Kit (Illumina, USA) following TruSeqTM RNA Sample Preparation Guide. Briefly, the poly-A containing mRNA molecules were purified using poly-T oligo-attached magnetic beads.

Following purification, the mRNA was fragmented into small pieces by incubation with divalent cations at 94 °C for 8 min. The cleaved RNA fragments were copied into first strand cDNA using reverse transcriptase and random primers, followed by second strand cDNA synthesis using DNA Polymerase I and RNase H. These cDNA fragments then underwent an end repair process; to add a single ‘A’ base and ligation of the adapters; before being purified and enriched with PCR to create the final cDNA library. Purified libraries were quantified by Qubit® 2.0 Fluorometer (Life Technologies, USA) and validated by Agilent 2100 bioanalyzer (Agilent Technologies, USA) to confirm the insert size and calculate the mole concentration. Cluster was generated by cBot with the library diluted to 10 pM and were then sequenced on the Illumina NovaSeq 6000 (Illumina, USA).

The library construction and sequencing was performed at Shanghai Sinomics Corporation.

### Quantitative Real-time reverse transcription (qRT-PCR)

ALI-hNECs were exposed to 25 μg/ml O_3_BC ± 200 μg/ml pollen in the absence or presence inhibitors (100 μM NAC, 10 μM MCC950, or 50 μM YVAD) for 24 h at 37 °C in CO_2_ incubator, and at the end of incubation total RNA was extracted from the cells using the TaKaRa MiniBEST Universal-RNA Extraction Kit (TAKARA BIO INC, Kyoto, JPN). The RNA was quantified using a NanoDrop 1000 spectrophotometer (Thermo Fisher Scientific, Carlsbad, CA, USA), and subjected to real-time PCR analysis using TB Green™Premix Ex Taq™II (TAKARA BIO INC, Kyoto, JPN) in a Bio-Rad real-time PCR detection system. GAPDH was used as an endogenous reference for mRNAs. All primers employed for PCR analysis were obtained from TSINGKE (TSINGKE Biological Technology, Beijing, CHN), as follows: GAPDH: sense 5ʹ-ACCACCATGGAGAAGGC-3ʹ, and antisense 5ʹ-GGCATGGACTGTGGT CATGA-3ʹ; IL-1β: sense 5ʹ-GTGGTGGTCGGAGATTCGTAG-3ʹ, and antisense 5ʹ-GAAATGATGGCTTATTACAGTGGC-3ʹ; CSF1: sense, 5ʹ-AGTATTGCCAA GGAGGTGTCAG-3ʹ, and antisense 5ʹ-ATCTGGCATGAAGTCTCCATTT-3ʹ; FCER1G: sense, 5ʹ-GAGAGCCTCAGCTCTGCTAT-3ʹ, and antisense 5ʹ-TGGTT ATAGCTGCCTTTCGCA-3. Relative expression was calculated using the comparative cycle threshold method.

### Statistical analysis

Data from at least three independent experiments were expressed as mean ± standard deviation (SD), and analyzed for significance using Spearman correlation coefficients, two-tailed indirect Student’s t-test or one-way analysis of variance (ANOVA), followed by the LSD post hoc test for multiple comparisons. All statistical analyses were performed using SPSS 25.0 statistical software and a P value < 0.05 was considered statistically significant.

The parameter Q value was used for statistical screening of differential genes. The difference screening criterion was q value < 0.05, and FC2, that is, the change of the expression value was up 2 times (FC ≥ 2) or down 2 times (FC ≤ 0.5). Enrichment analysis of different genes based on Gene Ontology (GO) and KEGG database for each Gene, was additionally performed to determine the major biological functions and pathways of the different genes [9, 10].

## Results

### ***Effect of O***_***3***_*** BC*** ± ***pollen on viability of hNECs***

**Fig. 1:**
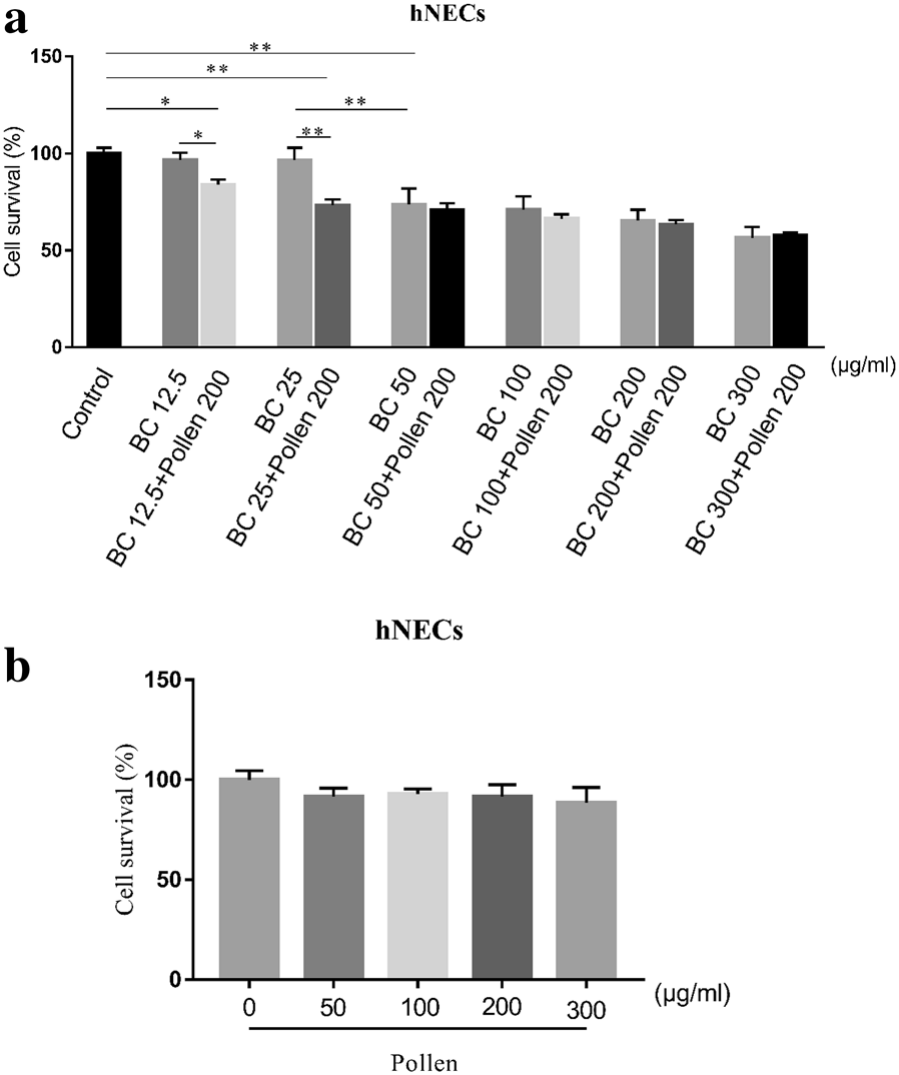
Effect of O_3_BC and pollen on the viability of hNECs. **a** Cell viability assay showing decreased viability in hNECs exposed to 50 μg/ml O_3_BC and 25 μg/ml O_3_BC + 200 μg/ml pollen; exposure to 25 μg/ml O_3_BC and 200 μg/ml pollen progressively decreased cell viability to 74.16 ± 2.74%, and the survival rate of cells treated with O_3_BC concentrations higher than 50 μg/ml was negatively correlated with the concentration of O_3_BC (r = − 0.850, p = 0.00001). **b** Cell viability assay showing no change in viability in hNECs exposed to different concentrations of pollen

Exposure to O_3_BC progressively decreased the viability of hNECs from concentrations above 50 μg/ml; with a concentration of 50 μg/ml significantly decreasing the viability to 73.57 ± 8.270%, compared to control and a O_3_BC concentration of 25 μg/ml (P < 0.01, P < 0.05, respectively). Furthermore, the survival rate of cells treated with O_3_BC concentrations higher than 50 μg/ml was negatively correlated with the concentration of O_3_BC (r = − 0.850, p = 0.00001) (Fig. 1a). In contrast, exposure to pollen alone did not alter the viability of hNECs at any concentration of pollen investigated (Fig. 1b). However, addition of 200 μg/ml pollen to O_3_BC 12.5 μg/ml or 25 μg/ml significantly decreased the viability of hNECs compared to control (viability = 83.56 ± 2.49%, P < 0.05; and 74.16 ± 2.74%, P < 0.01, respectively), as well as compared to O_3_BC 12.5 μg/ml or O_3_BC 25 μg/ml alone (P < 0.05 and P < 0.01, respectively) (Fig. 1a).

### Transcriptome alteration and functional enrichment analysis

**Fig. 2:**
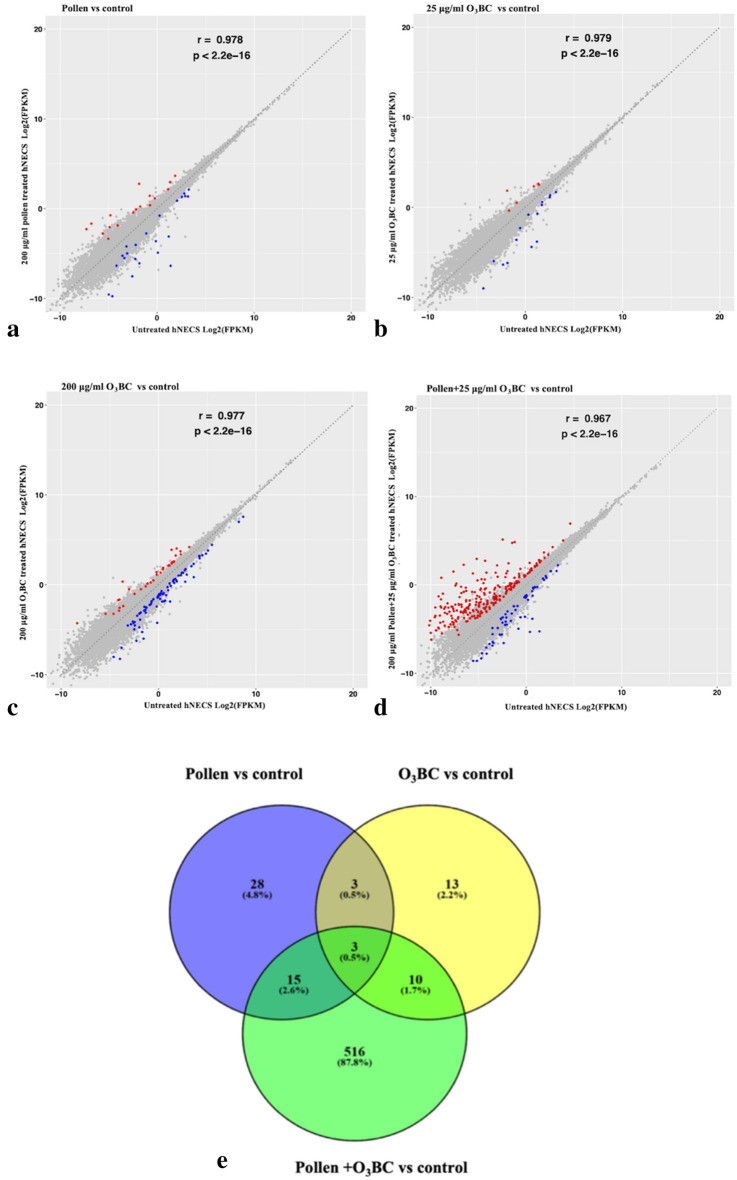
Transcriptomic alterations in hNECs treated with pollen, O_3_BC, or combination of pollen and O_3_BC. Scatter diagrams comparing the up-regulated (denoted in red) and down-regulated (denoted in blue) genes in untreated hNECS versus hNECs treated with **a** 200 μg/ml pollen; **b** 25 μg/ml O_3_BC; **c** 200 μg/ml O_3_BC; and **d** 25 μg/ml O_3_BC + 200 μg/ml pollen. **e** Venn diagram representing the numbers of overlapping regulated genes after treatment with 200 μg/ml pollen, 25 μg/ml O_3_BC, and 25 μg/ml O_3_BC + 200 μg/ml pollen, relative to control. q value < 0.05 and FC2

A subset of 58,300 human genes was assayed in this study. Gene expression changes were analyzed by comparison between pollen, O_3_BC, and O_3_BC+ pollen treated groups and the control groups, using criteria of > 2.0-fold changes with a p-value of < 0.05 being statistically significant. Exposure to 200 μg/ml pollen significantly altered expression of 49 genes (23 genes up-regulated and 26 genes down-regulated) (Fig. 2a), whereas exposure to 25 μg/ml and 200 μg/ml O_3_BC significantly altered expression of 29 genes (11 genes up-regulated and 18 genes down-regulated) (Fig. 2b), and 124 genes (38 genes up-regulated and 86 genes down-regulated) (Fig. 2c), respectively. Moreover, exposure to a combination of 25 μg/ml O_3_BC+ 200 μg/ml pollen resulted in significant alterations in even greater number of genes (467 genes up-regulated and 77 down-regulated) (Fig. 2d). Use of a Venn diagram demonstrated overlap of several differentially regulated genes relative to control, following exposure to 200 μg/ml pollen alone, 25 μg/ml O_3_BC, and 25 μg/ml O_3_BC+ 200 μg/ml pollen (Fig. 2e). Overall, 15 (2.6%) and 10 (1.7%) differentially regulated genes in 25 μg/ml O_3_BC+ 200 μg/ml pollen exposed cells overlapped with genes in pollen and O_3_BC alone exposed cells, respectively. In contrast, 516 differentially regulated genes (87.8%) in 25 μg/ml O_3_BC+ 200 μg/ml pollen did not overlap with any genes in pollen or O_3_BC alone exposed cells. These results indicated that there was significant difference in gene expression between pollen or O_3_BC alone and O_3_BC+ pollen exposed cells.

**Fig. 3:**
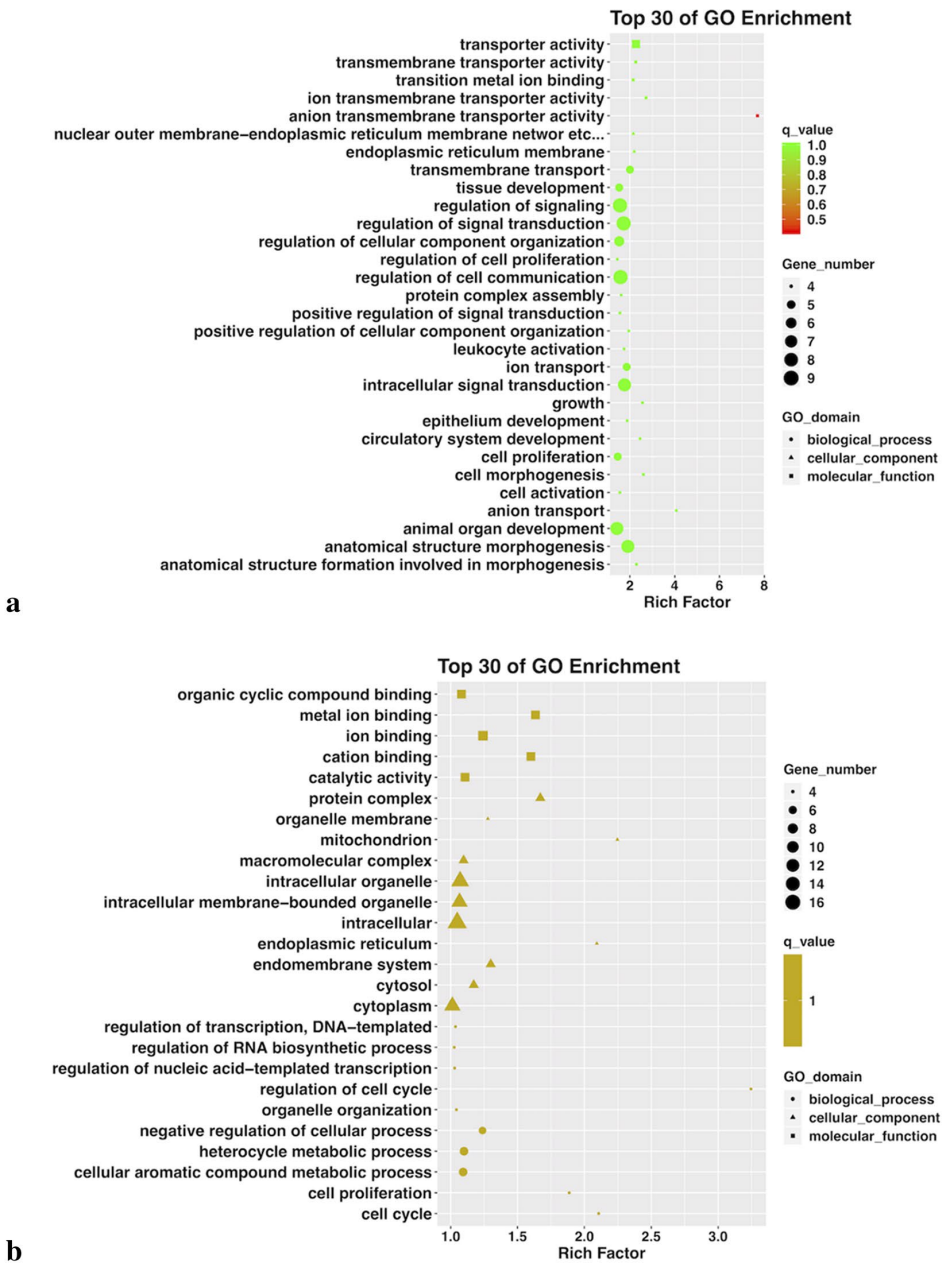

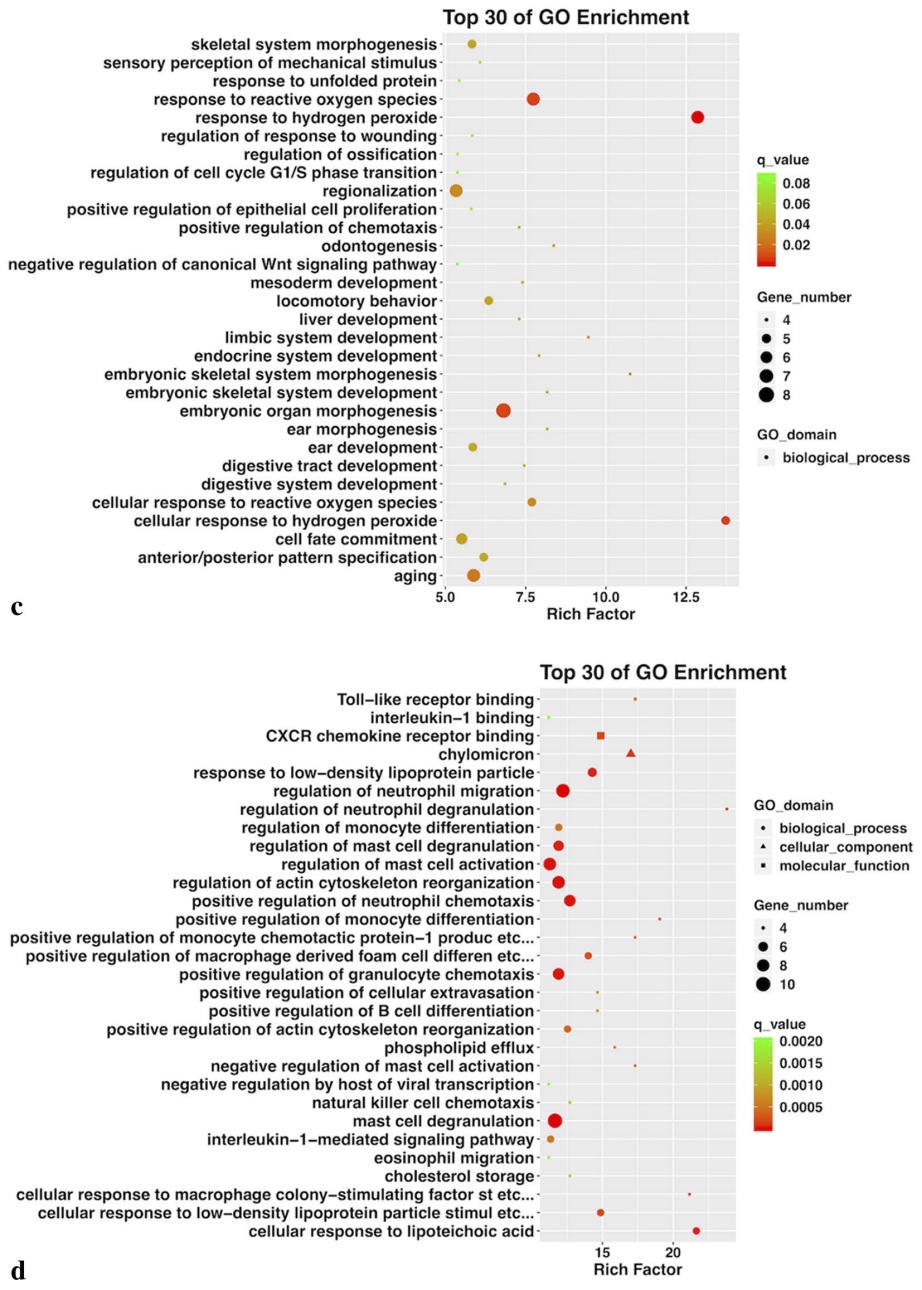
Gene ontology (GO) analysis by High-throughput RNA sequencing. Top 30 GO terms enriched by up-regulated and down-regulated genes in O_3_BC (25 and 300 μg/ml) or pollen treated hNECs. In each plot, the GO terms were aligned from left to right according to their Rich Factor from low to high. **a** 200 μg/ml pollen treatment, **b** 25 μg/ml O_3_BC treatment, **c** 300 μg/ml O_3_BC treatment, **d** 25 μg/ml O_3_BC + 200 μg/ml pollen treatment. q value < 0.05 and FC2

Gene ontology (GO) biological processes and KEGG pathway enrichment analysis of the altered genes further demonstrated that no GO terms were substantially enriched in hNECs exposed to 25 μg/ml O_3_BC or 200 μg/ml pollen alone (Fig. 3a, b). However, for hNECs exposed to 200 μg/ml O_3_BC, the up-regulated GO terms were mainly associated with hypoxia stress response (i.e., cellular response to oxygen species and response to hydrogen peroxide) (Fig. 3c); whereas the top enriched GO terms for hNECs exposed to a combination of O_3_BC (25 μg/ml) and pollen (200 μg/ml) were mostly associated with the inflammatory response (i.e., regulation of neutrophil migration and chemotaxis, macrophage differentiation and chemotaxis, mast cell activation, degranulation and mast cells medical immunity, and phagocytosis) (Fig. 3d).

Further investigation of the main altered genes associated with innate immune and inflammatory response in hNECs exposed to the combination of O_3_BC (25 μg/ml) and pollen (200 μg/ml) are shown in Table 1. In particular, differentially expressed genes associated with regulation of neutrophil migration and chemotaxis included IL1B, IL1R1, IL1A, ITGAM, CSF3R, S100A9, RAC2, CCL20, CXCL5, CXCL3, and CXCL8; Genes associated with macrophage differentiation and chemotaxis included CSF1, CSF1R, TLR2, HCLS1, MMP9, LPL, and CD36; genes associated with phagocytosis included IL1B, SLC11A1, ITGSAM, DOCK2, RAC2, CD14, CD36, PTPRC, and MERTK; and genes associated with regulation of mast cell activation, degranulation and mast cells-mediated immunity included FCER1G, FGR, LAT2, SYK, FES, RAC2, and VAV1.

**Table 1:** Differentially expressed genes associated with inflammatory response; including; regulation of neutrophil migration and chemotaxis, macrophage differentiation and chemotaxis, mast cell activation, degranulation and mast cells mediated immunity, phagocytosis, and cell response for lipoteichonic acid in hNECs exposed to 25 μg/ml O_3_BC + 200 μg/ml pollen vs control hNECs

Biological function	Related Gene	Log2FC	q-value	Biological function	Related Gene	Log2FC	q-value
Regulation of neutrophil degranulation, migration and chemotaxis	CCL20	8.81	5.01E−37		TLR2	1.27	3.71E−21
	ITGAM	7.72	8.65E−22	Regulation of phaocytosis	ITGAM	7.72	8.65E−22
	CXCL8	7.59	3.29E−68		DOCK2	6.53	5.20E−17
	IL1B	6.22	2.87E−56		IL1B	6.22	2.87E−56
	S100A9	5.99	7.24E−06		CD14	5.86	3.80E−14
	CXCL5	4.76	2.44E−06		PTPRC	5.55	9.34E−07
	IL1R1	3.63	7.47E−09		CD36	3.72	1.71E−10
	CSF3R	3.15	1.04E−05		SLC11A1	3.12	2.54E−14
	RAC2	3.05	2.40E−16		RAC2	3.05	2.40E−16
	CXCL3	2.01	1.51E−09		MERTK	2.82	1.21E−07
	IL1A	1.14	1.01E−07	Regulation of mast cells degranulation, activation and mast cells mediated immunity	VAV1	4.76	2.44E−06
Macrophage differentiation, chemotaxis	HCLS1	7.29	7.19E−19		FGR	4.43	1.89E−10
	MMP9	6.06	1.24E−48		FES	3.40	0.000367
	LPL	5.59	5.24E−07		RAC2	3.05	2.40E−16
	CSF1	4.23	4.08E−15		MILR1	1.87	0.01
	CD36	3.72	1.71E−10		LAT2	1.26	2.48E−07
	CSFR1	3.37	7.25E−18		FCER1G	1.21	8.12E−17

### KEGG pathway analysis

Assessment of the upstream regulators associated with inflammatory response in hNECs exposed to the combination of O_3_BC and pollen compared with control indicated that the top 10 upstream regulators were IL1B, CSF1, CCL2, TLR2, LPL, IGF8, SPP1, CXCL8, FCER1G and IL1RN (Table 2).

**Table 2:** upstream regulators associated with inflammatory response in hNECs exposed to 25 μg/ml O_3_BC+ 200 μg/ml pollen vs control hNECs

Upstream regulator	Expr Log Ratio	Activation z-score	p-value of overlap
IL1B	6.00	4.41	1.13E−14
CSF1	4.01	3.68	1.87E−12
CCL2	3.93	1.37	4.51E−10
TLR2	6.75	3.39	6.33E−08
LPL	3.88	0.71	1.67E−07
IGF8	6.47	2.01	6.48E−07
SPP1	10.13	2.74	1.101E−06
CXCL8	5.57	2.29	9.69E−08
FCER1G	3.21	2.11	0.0000018
IL1RN	7.89	− 2.61	3.07E−08

### Gene expressions validation by RT-qPCR analysis

**Fig. 4:**
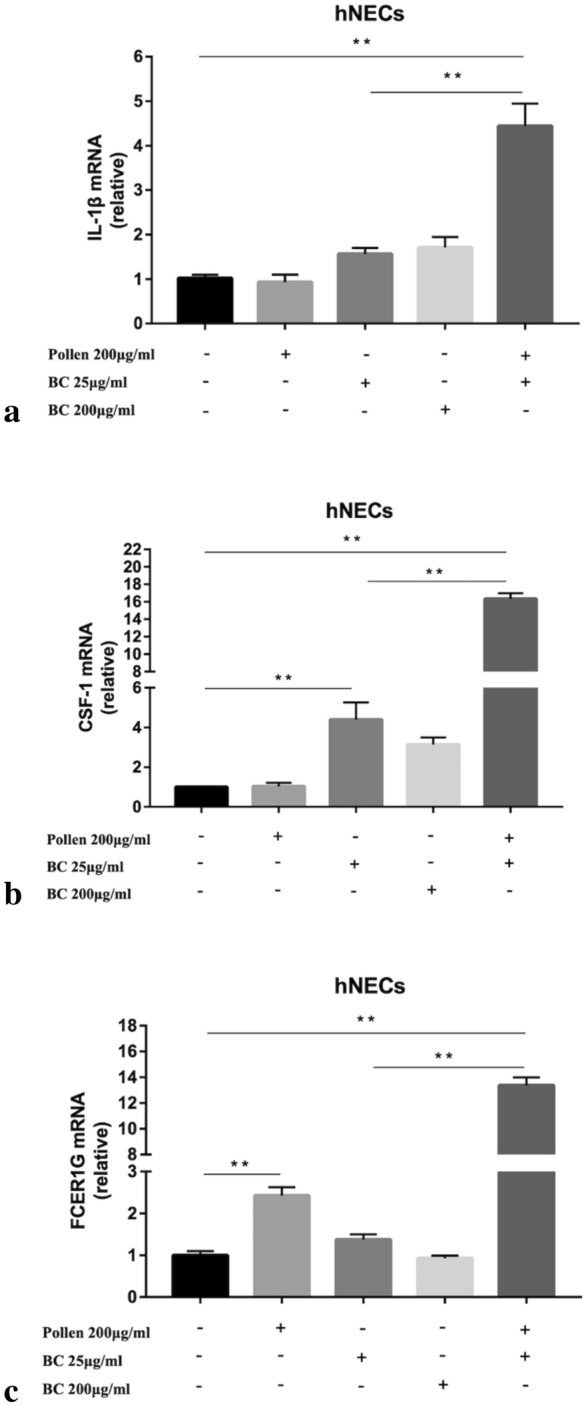
The expressions of inflammation related gene by RT-qPCR assay. IL1B, CSF1 and FCER1G were elevated in hNECs exposed to25 μg/ml O_3_BC + 200 μg/ml pollen as measured by RT-qPCR assay. n = 7, *p < 0.05, * *p < 0.01

In order to confirm the results of microarray, RT-qPCR was applied to further examine the expressions of target genes. Pro-inflammatory factor IL1B was up-regulated in hNECs exposed to 25, and 200 μg/ml O_3_BC and 25 μg/ml O_3_BC+ 200 μg/ml pollen (Fig. 4a). For inflammation related gene expressions, the primary regulator of mononuclear phagocytes, CSF-1 was over-expressed in hNECs exposed to 25 μg/ml O_3_BC and 25 μg/ml O_3_BC+ 200 μg/ml pollen; whereas for mast cell-mediated immunity related gene expressions, FCER1G was over-expressed in hNECs exposed to 200 μg/ml pollen and 25 μg/ml O_3_BC+ 200 μg/ml pollen. Although these results were consistent with the microarray data, there was a small difference in terms of the degree of over-expression between microarray method and RT-qPCR assay, which may due to differential sample preparation and sensitivity of the measuring method.

## Discussion

This study determined the effect of O_3_BC on pollen-sensitized human nasal epithelial cells (hNECs) and showed that exposure to more than 50 μg/ml of O_3_BC or 25 g/ml O_3_BC+ 200 g /ml pollen significantly decreased the viability of epithelial cells. Only a few genes were altered after exposure to 25 μg/ml O_3_BC or 200 μg/ml pollen allergen alone. Whilst exposure to 200 μg/ml O_3_BC, mainly up-regulated GO terms associated with hypoxia stress response, exposure to a combination of 25 μg/ml O_3_BC+ 200 μg/ml pollen upregulated GO terms related to innate immune and inflammatory responses, including regulation of neutrophil migration and chemotaxis, macrophage differentiation and chemotaxis, mast cell activation, degranulation and mast cells-mediated immunity, and phagocytosis. Furthermore, increased expression of genes IL (interleukin)1B, CSF1 and FCER1G were found to be the main upstream regulators involved in the inflammatory process.

IL1B is a potent proinflammatory cytokine, belonging to the IL-1 family cluster that includes the IL-1a, and IL1-RN genes. IL-1β can be activated by caspase 1 and induces neutrophil influx and activation, T-cell activation and cytokine production, B-cell activation and antibody production, and fibroblast proliferation and collagen production. Interleukin-1 receptor 1 (IL1R1) as a member of IL-1 family cluster may regulate the process of inflammation in organisms [11]. One recent study has reported that exposure to O_3_BC induced differential expression of IL-1R1 in A549 cells [12], which is consistent with the findings from the present study. Similarly, our findings for differential expression of genes in hNECs exposed to a combination of O_3_BC (25 μg/ml) and pollen (200 μg/ml), which influence neutrophil activity in terms of the top enriched GO terms, are consistent with other studies. For example, TGAM (integrin alpha M chain), a leukocyte-specific integrin, has been shown to be important in the regulation of neutrophil migration and phagocytosis-induced apoptosis in extravagated neutrophils [13]. Similarly, Rac2, a member of the Rho family GTPases, which are critical in regulating neutrophil activation, is involved in the control of the neutrophil actin cytoskeleton, cell migration, and the NADPH oxidase [14]. CSF3R (granulocyte colony-stimulating factor receptor) plays a crucial role in the proliferation, differentiation and survival of cells along the neutrophilic lineage. One of the most important functions of neutrophils is the production of oxidative metabolites for killing invading micro-organisms. Studies have suggested that S100A9 inhibits chemotaxis toward pro-inflammatory molecules and increased oxidative metabolism by neutrophils [15], which is consistent with our finding of the anti-oxidative and anti-inflammatory effect of S100A9 on neutrophils. An in vitro study has confirmed that CCL20 has a direct chemotactic effect in neutrophil recruitment [16]. In accordance with these studies, the present study has indicated that epithelium-derived neutrophil-activating peptide 78 (CXCL5), and neutrophil chemoattractants IL-8 (CXCL8) and CXCL3 are likely to be involved in the inflammatory response induced by O_3_BC and pollen in hNECs.

Macrophages are indispensable as members of the innate immune system, as they regulate normal physiology as first responders by communicating with the host’s adaptive immune system. Macrophage phenotypes include classically activated macrophages (M1) and alternatively activated macrophages (AAMφ or M2), of which M2 play a role in resolving inflammation. Canonical induction of M2 polarization is mediated by PPARs (peroxisome proliferator-activated receptors; PPARδ, PPARγ) and PGC1β (PPARγ coactivator-1 beta) activation, induction of cluster of differentiation 36 (CD36) and lipoprotein lipase (LPL). Several studies indicate that LPL is a primary regulator of macrophage lipid uptake and a modulator of macrophage polarity [17, 18]. Thus, the finding for increased expression of LPL in the present study would suggest that exposure to O_3_BC and pollen may induce macrophage polarization to M2. This is consistent with the finding for also increased expression of Colony-stimulating factor-1 (CSF-1, also known as macrophage-CSF); the primary regulator of the survival, proliferation, differentiation and function of mononuclear phagocytes; and CSF-1 receptor (CSF1R) following exposure to O_3_BC and pollen. Indeed, it has been demonstrated that CSF-1 enhances cytotoxicity, superoxide production, phagocytosis, chemotaxis and cytokine production in monocytes or macrophages when CSF-1 receptor (CSF1R) expressed on these cells is activated [19]. Similarly, CSF-1 can also prime some innate immune responses while suppressing others by modulation of Toll-like receptors (TLRs), such as TLR2, another gene found to be differentially expressed following exposure to BC and pollen. Moreover, our findings for differential expression of MMP-9 and HCLS1 are also in accordance with studies that have demonstrated that MMP-9 activation is important for macrophage migration, and HCLS1 enhances the function of monocytes/ macrophages [20].

Phagocytosis is primarily carried out by specialized cells termed professional phagocytes, which include cells of the immune system such as macrophages, neutrophils and dendritic cells. Of the differentially expressed genes associated with regulation of phagocytosis in the present study, pro-inflammatory cytokines IL1B (IL-1ß) and TNF-α, have been shown to upregulate Fc receptor-mediated phagocytosis [21]. SLC11A1 (Solute Carrier Family 11, Member 1), formerly known as natural resistance associated macrophage protein 1 (NRAMP) [22], is a member of the metal transporter protein family, which transfers iron (Fe) ions across the phagosome membrane [22]. Integrin ITGSAM is important in the phagocytosis of complement coated particles, and may regulate phagocytosis-induced apoptosis in extravagated neutrophils [23]. DOCK2 is a new member of the CDM family proteins, which plays an important role in phagocytosis and NADPH oxidation by functioning upstream of RAC2 [24]. Protein Tyrosine Phosphatase Receptor Type C (PTPRC, CD45) has been shown to be involved in phagocytosis as a positive regulator of Src family kinases (SFKs) [25]. CD36 and CD14 have been shown to interact with TLRs to induce phagocytosis and inflammation involving monocytes and macrophages [26, 27]. MERTK, a MER/AXL/TYRO3 receptor kinase family, plays a role in various processes such as macrophage clearance of apoptotic cells, platelet aggregation, cytoskeleton reorganization and engulfment [28].

The present study has demonstrated that exposure to O_3_BC and pollen also lead to differential expression of genes associated with regulation of mast cells. Mast cell mediator release plays a vital role in the initiation of inflammatory reactions associated with allergic disorders. This involves a chain of reactions following antigen-mediated aggregation of immunoglobulin E (IgE)-occupied high-affinity receptors for IgE (FceRI) on the mast cell surface; involving activation of the Src family tyrosine kinase (Syk, include Lyn and Fgr), phosphorylation of the transmembrane adapter molecules linker for activated T cells 1 (LAT1) and LAT2, activation of protein kinase C (PKC) and liberation of intracellular calcium. These signals lead to mast cell degranulation and contribute to activation of transcription factors required for cytokine and chemokine production. The role of LAT2 in mast cell activation is still enigmatic; however, it has been proposed to both upregulate and downregulate antigen-mediated responses, and to enhance FceRI-dependent degranulation [29]. In this pathway, tyrosine phosphorylation activates Vav1, a regulator of PLCγ-activated calcium signals to induce migration and activation of mast cells [30]. FGR positively regulates mast cell degranulation, production of eicosanoids and cytokines [31], and FES protein-tyrosine kinase, a downstream effector of KIT signalling in mast cells, is required for migration of mast cells.

However, a limitation of this study is that although we have previously confirmed the role of IL-1B in environmental black carbon exacerbated nasal epithelial inflammation [32] using both PCR and Western blot analyses, the findings for the changes in IL1B, CSF1, and FCeR1 gene expression noted by PCR analysis were not confirmed by Western blot analysis for protein expression of these mediators. Another limitation is that we only used O_3_BC and did not compare the effects of O_3_BC and BC on the hNECs.

## Conclusion

In summary, exposure to 25 g/ml O_3_BC and 200 g/ml pollen significantly decreases the viability of epithelial cells, alters expression of gene and induces innate immune and allergic inflammation in hNECs. IL1B, CSF1, and FCER1 are the main upstream regulators in the inflammatory response induced by O_3_BC and pollen.

## Data Availability

The datasets used and/or analysed during the current study are available from the corresponding author on reasonable request.

## Abbreviations

ALI cultures: Air–liquid interface cultures
AR: Allergic rhinitis
O_3_BC: Ozone-aged Black carbon
BCA: Bicinchoninic acid
BEGM: Bronchial epithelial growth medium
CSF-1(R): Colony-stimulating factor-1 (receptor)
DMEM: Dulbecco's modified eagle media
FceRI: High-affinity receptor for immunoglobulin E
GO: Gene ontology
hNECs: Human nasal epithelial cells
IgE: Immunoglobulin E
IL: Interleukin
IL1R1: Interleukin-1 receptor 1
KEGG: Kyoto encyclopedia of genes and genomes
LAT 1/2: Linker for activated T cells 1/2
LPL: Lipoprotein lipase
M1/M2: Classically activated macrophages/alternatively activated macrophages
NO_2_: Nitrogen dioxide
NRAMP: Natural resistance associated macrophage protein 1
PBS: Phosphate buffer saline
PM: Particulate matter
RNA-Seq: High-throughput RNA sequencing
PKC: Protein kinase C
PPAR: Peroxisome proliferator-activated receptor
PTPRC: Protein Tyrosine Phosphatase Receptor Type C
RT-qPCR: Reverse transcription-quantitative polymerase chain reaction
SFKs: Src family kinases
SLC11A1: Solute Carrier Family 11, Member 1
SO_2_: Sulphur dioxide
TER: Transepithelial resistance
TLRs: Toll-like receptors
